# Airway reactivity and sphingolipids—implications for childhood asthma

**DOI:** 10.1186/s40348-015-0025-3

**Published:** 2015-12-04

**Authors:** Jennie G. Ono, Tilla S. Worgall, Stefan Worgall

**Affiliations:** Department of Pediatrics, Weill Cornell Medical College, 505 East 70th Street, Box 211, New York, NY 10021 USA; Department of Pathology, Columbia University, New York, NY USA; Department of Genetic Medicine, Weill Cornell Medical College, New York, NY USA

**Keywords:** Sphingolipids, Asthma, Airway hyperreactivity, ORMDL3, Rhinovirus, SPT

## Abstract

Asthma is a clinically heterogeneous disorder, whose onset and progression results from a complex interplay between genetic susceptibility, allergens, and viral triggers. Sphingolipids and altered sphingolipid metabolism have emerged as potential key contributors to the pathogenesis of asthma. Orosomucoid-like 3 gene (ORMDL3) and the asthma susceptibility locus 17q21 have been strongly and reproducibly linked to childhood asthma, but how this gene is functionally linked to asthma is incompletely understood. ORMDL proteins play an integral role in sphingolipid homeostasis and synthesis, and asthma-associated ORMDL3 polymorphisms have been associated with early viral respiratory infections and increased risk of asthma. ORMDL proteins act as inhibitors of serine palmitoyl-CoA transferase (SPT), the rate-limiting enzyme for de novo sphingolipid synthesis, and decreased sphingolipid synthesis through SPT increases airway hyperreactivity, which is independent of allergy or inflammation. In allergic models of asthma, the sphingolipid mediators sphingosine-1-phosphate (S1P) and ceramide have been shown to be important signaling molecules for airway hyperreactivity, mast cell activation, and inflammation. This review will highlight how sphingolipids and altered sphingolipid metabolism may contribute towards the underlying mechanisms of childhood asthma.

## Introduction

Asthma is a chronic airway disease characterized by reversible airway obstruction, chronic inflammation, mucous production, and airway hyperreactivity. Asthma is a common and clinically heterogeneous disorder and poses huge costs to society [1]. The risk for asthma is determined in infancy and childhood, it is highly heritable, and the phenotypes are conferred by both genetic susceptibility and environmental exposures [2, 3]. Asthma exacerbations are triggered by environmental stimuli, most often respiratory viruses and allergens. Allergic sensitization commonly occurs in children with asthma, although up to half of those with mild to moderate disease will be non-allergic [4] and will respond poorly to current therapies which focus primarily on the inflammatory and allergic components of the disorder [5]. The variation in phenotypes suggests distinct underlying pathophysiology, and asthma is increasingly being viewed as a syndrome rather than a single disease [3].

For most asthma types, a genetic predisposition is present and essential for the “asthmatic reaction” to environmental stimuli. Over 100 genes have been identified in association with asthma [2]; among them, the orosomucoid-like 3 gene (ORMDL3) and the associated 17q21 locus have emerged through genome-wide association studies as likely contributors to the genetic susceptibility and underlying pathogenesis of asthma. While the functions of ORMDL3 are incompletely understood, it is known to be involved in sphingolipid metabolism and de novo sphingolipid synthesis [6], suggesting altered sphingolipid metabolism as a contributing factor in asthma. Ceramides and sphingosine-1-phosphate (S1P) have been the most extensively studied sphingolipids and are important bioactive signaling molecules [6, 7]. In addition to their role in asthma, sphingolipids have been associated with other pulmonary disorders including chronic obstructive pulmonary disease (COPD)/emphysema, cystic fibrosis, vascular permeability, and acute lung injury [8].

## Review

### Sphingolipids and asthma

**Fig. 1:**
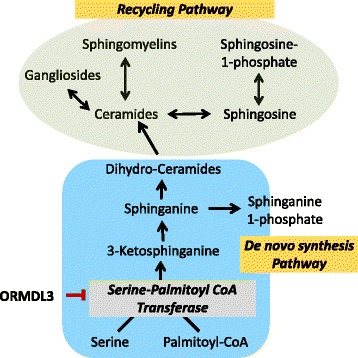
Sphingolipid synthesis. *Highlighted* are de novo and recycling pathways of sphingolipid metabolism and the alleged inhibitory effect of ORMDL3 on SPT

Sphingolipids are a diverse and complex category of lipids due to their numerous variations in their sphingoid bases, fatty acids, and head groups [6, 7, 9]. They are key structural elements in cellular membranes and are essential signaling molecules for a wide range of cellular functions including growth and differentiation, signal transduction, immune response, cell proliferation, and apoptosis [7]. Acylation of the sphingoid backbone by specific ceramide synthases yields different ceramides, which vary by acyl chain length. Ceramide serves as a substrate for the production of complex sphingolipids, including sphingomyelin and glycosphingolipids. Ceramide can be generated via de novo sphingolipid synthesis or can be regenerated from hydrolysis of complex sphingolipids through the recycling pathways [7, 9, 10] (Fig. 1).

Most studies of sphingolipids and asthma have focused on inflammatory and allergic mechanisms related to the sphingolipid mediator S1P [11–19]. S1P is derived from sphingosine through phosphorylation by two sphingosine kinases (SphK1 and SphK2) which are widely expressed, including in bronchial epithelium and airway smooth muscle cells [20]. Through the activation of different signaling pathways, S1P mediates a diverse set of biological processes, acting as both an intracellular second messenger and as an extracellular ligand for specific cell surface G protein-coupled receptors, S1P_1_–S1P_5_ [21]. S1P and SphK have been implicated in airway smooth muscle cell hyperresponsiveness, lung inflammation, and mast cell activation, all key features in the pathogenesis of asthma. S1P and the SphK pathways have therefore been targeted for the development of sphingolipid-based therapeutic agents, though the role of S1P and its receptors remains incompletely understood. For example, the immunomodulating agent FTY720 (Fingolimod), approved for the treatment of multiple sclerosis, attenuates allergen-induced inflammation, as well as airway hyperreactivity in mouse models of asthma [12, 22]. This effect was also shown with *N*,*N*-dimethylsphingosine (DMS), an SphK inhibitor [15, 20]. Neither of these has been tested for clinical use in asthma yet.

In the mouse, exogenous systemic administration of S1P resulted in increased contraction of the bronchi, increased airway resistance, as well as mast cell and eosinophil recruitment to the lung [18], and enhanced methacholine-induced contractions in guinea pig tracheal smooth muscle [23]. S1P has also been shown to be important in immunoglobulin E (IgE)-mediated mast cell migration and degranulation [24], allergic asthma, and secretion of pro-inflammatory cytokines [25]. Mast cells play a central role in the development of asthma, and cross-linking of FCεR_1_, the high-affinity IgE receptor, induces SphK activation and S1P secretion [24, 26].

In humans, S1P levels are significantly increased in bronchioalveolar lavage (BAL) fluid from subjects with asthma following segmental allergen challenge compared to control subjects [11]. Decreased protein levels for S1P_1_ receptor have been demonstrated in adults with asthma, and polymorphisms in S1P_1_ have been associated with an increased risk for asthma [19]. In addition, ceramide (C16) levels were noted to be increased in the exhaled breath collection of seriously ill subjects with asthma, compared to healthy controls [22].

Ceramides have also recently been implicated in the pathogenesis of COPD and emphysema. Although not relevant for the pediatric population, Asthma-COPD Overlap Syndrome (ACOS), a poorly identified but increasingly recognized entity, could provide some further clues for the role of sphingolipids in airway diseases. In a joint statement, ACOS has recently been recognized by the Global Initiative for Asthma (GINA www.ginasthma.org) and the Global Initiative for Chronic Obstructive Lung Disease (GOLD www.goldcopd.org) as a distinct clinical entity, encompassing individuals who have clinical symptoms that are characteristic of both asthma and COPD [27]. Like asthma, COPD is characterized by obstruction due to smooth muscle contraction, increased mucus production, and chronic inflammation [27, 28]. Also like asthma, COPD is a heterogeneous disorder with variable clinical phenotypes which is impacted by environmental factors [27, 28]. Cigarette smoke exposure is a major risk factor in developing this disease; however, most smokers do not develop COPD [28] suggestive of an underlying susceptibility to this environmental insult in those patients. Altered sphingolipids and sphingolipid metabolism has been suggested as a possible mechanism in this susceptibility [29].

Lung ceramide levels where shown to be higher in human subjects with emphysema (a specific phenotype of COPD) compared to those without [30], and the expression of multiple species of ceramides, dihydroceramides, glycosphingolipids, and sphingomyelins were shown to be significantly higher in smokers with COPD than those in non-smokers [29]. In another recent study looking at the association between sphingolipid species and different COPD phenotypes, plasma sphingolipids were shown to be inversely related to emphysema severity and positively associated with severe COPD exacerbations [31].

### ORMDL3 and asthma

ORMDL3 on chromosome 17 (17q21) has been strongly and consistently linked to asthma in multiple ethnic groups [32–41]. Single nucleotide polymorphisms (SNPs) within the 17q21 asthma susceptibility locus achieved genome-wide level significance with childhood-onset asthma [42] and have been since widely replicated. Moffat et al. showed that in Epstein-Barr virus-transformed lymphoblastoid cell lines, transcript levels of ORMDL3 were positively associated with rs7216389, the SNP with the strongest association with childhood asthma [38]. This suggested that variants at this asthma susceptibility locus may regulate ORMDL3 expression, having also been confirmed in rhinovirus-infected blood cells [43].

Polymorphisms at the ORMDL3 locus have been associated with increased risk for asthma [34, 44–46], severe asthma [32, 47], and early viral respiratory infections in asthma [40]. Infection with respiratory viruses is a well-known risk factor for persistent wheezing and a risk factor for asthma later in life [40, 43]. Variants at the 17q21 locus are shown to enhance the association between early respiratory infections and childhood asthma [37, 40, 43]. In particular, infections with human rhinovirus (HRV), the most common trigger of asthma exacerbations [48, 49], are associated with a more than 10-fold increased odds ratio for childhood asthma in children who carry the asthma-associated ORMDL3 genotype [43]. Interestingly, this effect was not seen in association with respiratory syncytial virus (RSV), a commonly associated virus with early-onset wheezing and bronchiolitis in infants and children [43]. Although infection with HRV is associated with increased risk for the development of asthma [40, 43, 50], only a portion of children exposed go on to develop the disease suggesting that the host genotype likely plays a role [43]. In another study, Smit et al. showed that the association between early viral infection and asthma had a greater than twofold difference in odds ratio in individuals who were homozygous for the risk-related alleles at the ORMDL3-associated SNPs [40]. This association was further enhanced when children with risk-related variants were exposed to tobacco smoke in early life [34, 40].

In mouse lungs, ORMDL3 expression can be increased by a variety of stimuli, such as allergens, tobacco smoke, and lipopolysaccharides [51]. Although the 17q21 polymorphisms which control ORMDL3 expression have not been associated with atopy [42, 45, 52], some seem related to T helper cell type 2 (Th2) cytokine responses [53] and asthmatic responses to allergens [54]. Overexpression of human ORMDL3 in transgenic mice showed an associated increase in airway remodeling (smooth muscle, fibrosis, mucous production) and an enhanced IgE response compared to wild-type mice following allergen challenge [55].

Though the underlying mechanisms functionally linking ORMDL3 to asthma remain largely unknown, a growing body of evidence supports ORMDL3 as contributing to the etiology of asthma, where it likely participates in multiple pathways important to its underlying pathogenesis. In mice, ORMDL3 was shown to be an allergen and Th2 cytokine-inducible gene which regulates the expression of chemokines, metalloproteinases, and oligoadenylate synthetases through activation of the unfolded protein response (UPR). This led to epithelial cell remodeling through its effect on the sarco/endoplasmic reticulum Ca-ATPase (SERCA) [51, 55]. In contrast, a study in human airway epithelial cells did not find inflammation or UPR activation to be associated with ORMDL3 expression [56]. ORMDL3 has also been implicated in endoplasmic reticulum-mediated calcium signaling and stress responses in immune cells [57], as well as in eosinophil trafficking, recruitment, and degranulation in murine models of allergic asthma [58].

### ORMDL3, asthma, and sphingolipid metabolism

Another potential mechanism linking ORMDL3 to asthma is through alterations in sphingolipid homeostasis and de novo sphingolipid synthesis. The de novo pathway of sphingolipid synthesis which originates in the endoplasmic reticulum (ER) is a key mechanism for regulating cellular levels of ceramide and other sphingolipids [7]. ORMDL proteins act as inhibitors of serine palmitoyl-CoA transferase (SPT), the rate-limiting enzyme for de novo sphingolipid synthesis [59–61], and regulate cellular ceramide levels [22, 61, 62]. ORMDL3 is localized to the ER and is highly expressed in airway epithelial cells [51]. The first step in de novo sphingolipid synthesis begins with the condensation of serine and palmitoyl-CoA by SPT. The reaction product, 3-ketosphinganine, is unstable and is rapidly converted to sphinganine. Sphinganine is further metabolized by distinct ceramide synthases to dihydroceramides which can then generate ceramides via dihydroceramide desaturase [10]. ORMDLs have been shown to mediate the regulation of sphingolipid homeostasis in response to overall cellular sphingolipid levels and maintain physiologic sphingolipid concentrations in the face of external perturbations [6, 59, 61–63].

Alteration in de novo sphingolipid synthesis has emerged as a contributing factor in airway hyperreactivity, a cardinal feature of all asthma types. Given that the asthma-associated ORMDL3 polymorphisms lead to increased expression of ORMDL3 [38], it has been suggested that asthma-associated SNPs negatively regulate SPT resulting in inhibited de novo sphingolipid synthesis [64]. How ORMDL3 regulates SPT activity remains largely unknown; however, ORMDL has been shown to form stable complexes with SPT [59, 61], and ORMDL3 expression is dependent on and responsive to the overall SPT activity of the cell [62, 65].

The regulation of sphingolipid metabolism is complex, incompletely understood, and involves a network of multiple interconnected mechanisms [6, 61]. Knockdown of ORMDL1, 2, and 3 in mammalian cells has been shown to increase ceramides [22, 59, 61]; however, the overexpression of ORMDL3 appears to have a differential response. In airway epithelial cell lines, a modest overexpression of ORMDL3 decreases ceramide levels likely due to inhibition of SPT-dependent de novo synthesis. However, robust overexpression of ORMDL3 resulted in overall increased ceramide levels [22, 62] suggesting a possible contribution of the recycling/salvage sphingolipid synthesis pathways [22] and indicating that relative cellular concentrations of SPT and ORMDL are important in the regulation of de novo sphingolipid synthesis by ORMDL expression [62].

In mouse models, airway hyperreactivity has been associated with alterations in de novo sphingolipid synthesis through effects on ORMDL and SPT. In a transgenic mouse model, overexpression of human ORMDL3 showed increased airway responsiveness to methacholine challenge compared to wild-type mice [55]. Decreased de novo sphingolipid synthesis either by direct pharmacologic inhibition with myriocin or genetic haploinsufficiency of SPT increases airway reactivity in the absence of allergic sensitization or inflammation [64]. Increased hyperreactivity was also noted in isolated human bronchial rings which underwent SPT inhibition by myriocin [64]. In an allergic mouse model, inhibition of SPT with myriocin was also associated with the exacerbation of airway hyperreactivity, in recent work by Edukulla and Lindsley [66]. Although the regulation of sphingolipid metabolism is clearly complex, these findings suggest altered sphingolipid homeostasis as an important contributor for asthma pathogenesis [67] and remains an active area of ongoing research.

## Conclusions

Sphingolipids, long known as important structural components of the cell, have emerged as important cell signaling molecules regulating a wide range of cellular functions, including cell proliferation and apoptosis, vascular and epithelial integrity, cell contact and adhesion, innate and acquired immunity, and inflammation [6, 7, 68, 69]. The role of sphingolipids and their effects on airway function and disease are highly integrated and complex; though in its early stages, our understanding of these roles is rapidly expanding. The sphingolipid metabolites S1P and ceramides have been implicated in the pathogenesis of pulmonary diseases, including asthma. In addition, alterations in de novo sphingolipid metabolism have been shown to lead to airway hyperreactivity, the cardinal feature of asthma, without allergic sensitization or inflammation. Sphingolipid synthesis thus represents a novel metabolic pathway influencing airway smooth muscle contractility, and strategies to affect sphingolipid homeostasis and metabolism hold promise as novel and more personalized approaches to treat childhood asthma in the future.

## Abbreviations

COPD: chronic obstructive pulmonary disease
HRV: human rhinovirus
IgE: immunoglobulin E
ORMDL3: orosomucoid-like 3 gene
S1P: sphingosine-1-phosphate
SNP: single nucleotide polymorphism
SPT: serine palmitoyl-CoA transferase
Th2: T helper cell type 2
